# Secondary Terpenes in *Cannabis sativa* L.: Synthesis and Synergy

**DOI:** 10.3390/biomedicines10123142

**Published:** 2022-12-06

**Authors:** Francisco T. Chacon, Wesley M. Raup-Konsavage, Kent E. Vrana, Joshua J. Kellogg

**Affiliations:** 1 Intercollege Graduate Degree Program in Plant Biology, Pennsylvania State University, University Park, State College, PA 16802, USA; 2 Department of Pharmacology, Penn State College of Medicine, Hershey, PA 17033, USA; 3 Department of Veterinary and Biomedical Sciences, Pennsylvania State University, University Park, State College, PA 16802, USA

**Keywords:** cannabis, phytochemistry, cannabinoids, terpenes, synergy, entourage effect, biosynthesis

## Abstract

Cannabis is a complex biosynthetic plant, with a long history of medicinal use. While cannabinoids have received the majority of the attention for their psychoactive and pharmacological activities, cannabis produces a diverse array of phytochemicals, such as terpenes. These compounds are known to play a role in the aroma and flavor of cannabis but are potent biologically active molecules that exert effects on infectious as well as chronic diseases. Furthermore, terpenes have the potential to play important roles, such as synergistic and/or entourage compounds that modulate the activity of the cannabinoids. This review highlights the diversity and bioactivities of terpenes in cannabis, especially minor or secondary terpenes that are less concentrated in cannabis on a by-mass basis. We also explore the question of the entourage effect in cannabis, which studies to date have supported or refuted the concept of synergy in cannabis, and where synergy experimentation is headed, to better understand the interplay between phytochemicals within *Cannabis sativa* L.

## 1. Introduction

*Cannabis sativa* L. is a dioecious plant of the Cannabaceae family and is perhaps most famous for its production of the psychedelic metabolite delta-9 tetrahydrocannabinol (D9-THC). Cannabis has been used in traditional medicine for millennia across several continents; cannabis has been used in traditional Chinese medicine therapies for the treatment of gout, pain, convulsions, insomnia, cough, headache, itching, and anemia [1], while in traditional Aryuvedic practices, cannabis has been reported to stimulate digestion, function as an analgesic and sedative, and have aphrodisiac, anti-parasitic, and anti-viral properties [2]. Review articles covering the chemistry, pharmacology, botany, genomics, and ethnology of cannabis are regularly published as the plant’s usage grows in prevalence [3,4,5,6,7]. In addition to THC, cannabis produces a number of other cannabinoid compounds with potent activities. Cannabidiol (CBD) is one non-psychedelic cannabinoid that has emerged as a popular botanical supplement ingredient [8]. A majority of Americans are aware of CBD, and ca. 18% have tried or are regular users of CBD products [9]. The US hemp-derived market in cannabidiol (CBD) topped $4.7 billion in 2021 and is expected to reach $12.0 billion by 2026 [10]. However, while many bioactivities can be ascribed to the presence of cannabinoids, cannabis is a prolific biosynthetic organism, producing over 750 known phytochemicals, including flavonoids and terpenoids, many of which possess putative medicinal properties [11], yet the majority of these phytochemical constituents and their mechanisms of action have not been fully explored.

Terpenes (also termed isoprenoids) are the most diverse class of natural products and are the most abundant by mass [12]; in cannabis, terpenes account for 3–5% of the dry mass of the inflorescence [13]. Terpenes have incredible potential for bioactivity against both infectious and chronic health conditions [14,15,16] and have been employed for thousands of years for therapeutic purposes, including in anti-inflammatory, anti-microbial, antioxidant, antitumor, and antidiabetic capacities [17]. In addition, terpenes often provide the foundation for the flavor and aroma of numerous plants and food products [18,19,20], including cannabis [21], granting the plant earthy or herbal aromas that combine with hints of sweet, citrusy, or piney scents. The terpene profile and content of cannabis has been reviewed previously [13,22,23]; however, analytical profiling studies, as well as cannabis phytochemistry reviews, traditionally focus on the more prevalent, terpenes such as myrcene, α-pinene, limonene, β-caryophyllene, linalool, humulene, ocimene, bisabolol, and terpinolene. The presence of a vast array of terpenes highlights the additional complexity of cannabis, as well as the further potential for bioactivity within this complex plant.

In botanical samples, mixtures of phytochemicals are often more effective than their individual constituents in isolation due to additive or synergistic interactions among compounds. Indeed, many chronic and infectious diseases are not regulated by a single cellular target, but often have multiple regulating pathways [24,25]. As organisms in a complex and dynamic ecological environment, plants have evolved to address this multifactorial disease etiology through the synthesis of structurally and functionally diverse phytochemicals. Thus, cannabis may also exert its bioactive effects via a combination of multiple constituents. Originally hypothesized in the late 20th century and termed the “entourage effect” [26], synergy between different cannabinoids has been documented in several studies. However, the potential for synergy between cannabinoids and other chemical classes, especially terpenes, has remained underreported.

This review aims to synthesize recent studies and information regarding the compositional diversity of terpenes, especially ‘minor’ terpenoid structures (compounds that are less prevalent in the plant on a by-mass basis) that have not been the focus of other reviews, yet are found in diverse cultivars of cannabis and have unique and varied bioactivities as well. This is a unique feature of this review. In addition, we will build on the body of knowledge regarding how terpenes can potentially work in concert with cannabinoids to enhance bioactivity, as this is a timely topic given the upswing in interest in cannabis and potential synergy/entourage effects.

## 2. Terpene Biosynthesis

**Figure 1:**
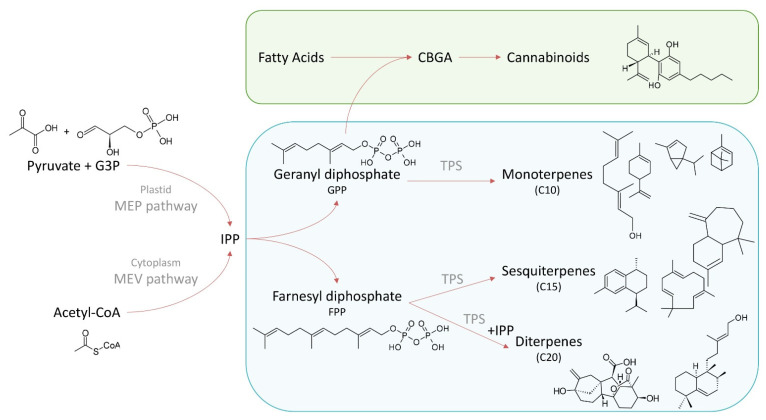
General scheme of terpene synthesis pathway in *Cannabis sativa* L.

Terpenes originate from the 5-carbon precursor isopentenyl diphosphate (IPP), which is biosynthesized from either pyruvate and glyceraldehyde (via the methylerythritol phosphate (MEP) pathway in plastids) [27] or from acetyl-coA (via the mevalonic acid (MEV) pathway in the cytoplasm) [28] (Figure 1). One or more IPPs condense with dimethylallyl diphosphate (DMAPP) in a 1′–4 fashion to form geranyl diphosphate (GPP, C10), farnesyl diphosphate (FPP, C15), or geranylgeranyl diphosphate (GGPP, C20). GPP and FPP serve as substrates for a multitude of synthetic reactions, condensing together to form the precursors of carotenoids and steroids, or cyclizing to form a myriad of terpene natural products (e.g., monoterpenes (C10), sesquiterpenes (C15), and diterpenes (C20)) [12,29]. GPP also condenses with a diphenol with an alkyl chain (e.g., olivetolic acid) to form the cannabinoids [30]. In cannabis, over 200 terpenes have been published to date [31].

Terpenoid biosynthesis is governed by a family of homologous enzymes, the terpene synthases (TPS) [29,32], which catalyze the formation of different types of terpenes, including monoterpenes, diterpenes, hemiterpenes, and sesquiterpenes. These essential enzymes are encoded in large gene families that have been broken down into seven subfamilies based on phylogenetic analyses rendering, TPS-a, -b, -c, -d, -e/-f, -g, and -h, each based on amino acid length and location of emergence, such as angiosperms or gymnosperms [29,33]. In angiosperms, the TPS-a subfamily contains sesquiterpene synthases (sesqui-TPSs); the TPS-b subfamily contains monoterpenes synthases (mono-TPSs) and hemiterpene synthases [34].

Booth et al. analyzed the genome and transciptome of Purple Kush cannabis to identify more than 30 cannabis terpene synthases (CsTPS genes) [35], which has been expanded to over 14 cultivars, representing chemotypes I, II, and III [34,36,37]. The characterized TPS genes of cannabis are documented as being a part of the TPS-a and TPS-b subfamilies [29]. Only nine of the 30 CsTPS genes have been fully characterized with respect to their catalytic functions, eight of which are multi-product enzymes that can generate different terpene structures from either GPP or FPP substrates [35,38]. Interestingly, genetic variation in these CsTPS has been associated with differences in the Sativa-Indica scale of cannabis labeling. Genotyping 100 cannabis samples for >100,000 single nucleotide polymorphisms revealed that Sativa- and Indica-labelled samples were indistinguishable from a genome perspective; however, variation in CsTPS genes translated to shifts in the terpene profile and was correlated with the current dichotomous label system, suggesting terpenes (and genetic markers associated with terpene biosynthesis) could have a large role in governing the strain classification [39]. This biosynthetic plasticity could be one explanation for the diversity of terpenes found in cannabis; however, it is important to keep in mind that the CsTPS responsible for many cannabis terpenes remain unexplored. When considering the incredible diversity of cannabis terpenes, it is unknown how the expression levels of different CsTPS could vary with plant development stage, plant organ and cell-type, and environmental factors. In addition, non-enzymatic modifications of terpenes, such as cyclization and oxidation, can increase structural diversity independent of enzymatic biochemical reactions. Even post-harvest considerations can change the terpene profile, especially the smaller, more volatile hemiterpenes and monoterpenes [40]. More qualitative and quantitative studies are needed to comprehensively profile the terpenes found in cannabis and how those concentrations relate to expression levels and functionality of the CsTPS.

## 3. Terpene Diversity in Cannabis

Over 20,000 terpenes have been identified in the Plantae kingdom, making these highly volatile compounds one of the most structurally and functionally diverse groups of natural products [41]. Cannabis is widely known for its assorted terpene profiles. To date, 200 terpenes/terpenoids have been detected in cannabis [42]. However, the complete identification and quantification of the vast majority of terpenes/terpenoids remains undetermined, blunting our knowledge of the impact of cannabis terpenes on plant and human health [43]. Thus, the complete identification of terpenes in cannabis may suggest a substantial assortment of cannabis terpenes unknown to current breeders and researchers.

With the tremendous diversity of compounds in cannabis, researchers seek to categorize the main chemical constituents of cannabis cultivars or ‘strains’ by establishing five classes of chemotypes based on cannabinoid ratios. These are classified as Chemotypes (I): high THCA:CBDA ratio; (II) intermediate ratios of THCA:CBDA; (III) low THCA:CBDA ratio; (IV) high CBGA content/low ratio of THCA:CBDA; and (V) containing almost no cannabinoids [44]. This classification has drawn researchers to further categorize cannabis chemical profiles by associating cannabinoid content with bioactive metabolites such as terpenes. Table 1 illustrates the concentration range (mg/g) of terpenes and terpene derivatives reported in published research articles investigating the terpene content of specific cannabis chemotypes. Chemical profiles of common cannabis cultivars continue to show that myrcene, β-caryophyllene, limonene, α-terpinene, and α-pinene are the most prominent terpenes that can be found in the first three chemotype varieties [34,45,46,47,48]. Terpene profiles of the remaining chemotypes are limited or have yet to be investigated. Conversely, the classification of secondary terpenes (terpenes found in lower concentrations) in cannabis chemotypes is limited, as they are often disregarded or unreported due to a lack of reference material. More studies on cannabis terpene chemotypes are required to identify the relationships between specific terpenes and cannabinoid content.

Birenboim et al., 2022, were the first to demonstrate a highly accurate classification of medicinal cannabis chemovars based on their cannabinoid and terpene profiles. Using a partial least-square discriminant analysis multivariate (PLS-DA) technique, Birenboim et al. were able to differentiate terpene content between the inflorescences of three major chemovars (high-THCA, high-CBGA, and a hybrid). They concluded that the terpenes of the three major classes were significantly different in their concentrations of different terpenes [49], providing evidence of the high-THCA class having a higher abundance of limonene, β-caryophyllene, β-pinene, α-humulene, γ-elemene, and seychellene. Within the hybrid class, α-pinene and β-myrcene are more pronounced, followed by a high abundance of γ-eudesmol, α-bisabolol, and guaiol in the high-CBGA class. However, these results represent 14 different cannabis chemovars, including seven high THC chemovars, five hybrid chemovars, and only two high-CBG chemovars. The plant material used was from commercial breeding lines that could not be affiliated to a specific subspecies because of crossings between different cultivars over many generations. Moreover, several factors have been shown to influence terpene diversity, such as plant genetics, pest presence, overall plant health, soil composition, proper drying, curing, and microbiology [34,50,51,52,53,54,55].

Variations in terpene expression can also be dependent upon the stage of growth. In 2016, Aizpurua-Olaizola et al. analyzed the terpene and cannabinoid content of the leaves and flowers of cannabis chemotypes I, II, and III. For 23 weeks, a chemical profile was generated on a weekly basis, providing the researchers with a total content of cannabinoids and terpenes at different stages of growth. Researchers found that chemotypes II and III required more time to reach their peak production of monoterpenes compared to chemotype I. Major terpene differences were also observed between chemotypes I and III. The distinct terpenes of chemotype I included γ-selinene, β-selinene, α-gurjunene, γ-elemene, Selina-3.7 (11) diene, and β-curcumene, while chemotype III displayed β-eudesmol, γ-eudesmol, guaiol, α-bisabolol, or eucalyptol. This suggests a chemotype-dependent terpene distribution, as the investigators describe the more prominent terpenes in chemotype III as having a higher correlation coefficient with CBDA and chemotype I terpenes having a higher correlation coefficient with THCA [53]. Despite the differences in terpene content at different stages of growth, limitations of terpenes and cannabinoid expression may be observed based on light exposure and select spectra.

A high abundance of terpenes and cannabinoids can be found on the surface of cannabis inflorescence and leaves in the glandular appendages known as trichomes [56,57]. Trichomes are believed to be a defense mechanism against several different stresses, including light stress [58,59]. This has led to the proposed ecological function of cannabinoids and terpenes aiding in protection against high light exposure [58]. Additionally, research has shown the altering effects LED light can have on THC and terpene concentrations, but not CBD [57,58]. One study provided evidence of supplemental green light increasing THC and terpene content in comparison to controls. However, quantification of IPP and DMAPP were not conducted, leaving the mechanistic implications undetermined [52]. With the increasing application of LED lighting for indoor cultivation, the chemical profiles of the desired chemotype may be susceptible based on light application. Nonetheless, with the information surrounding the factors that influence terpene concentrations, terpene biosynthesis, and genetic expression, new cultivars with desired cannabinoid and terpene profiles may become attainable as the research surrounding terpenes in cannabis continues.

**Table 1:** Concentrations of terpenes found in cannabis. Concentration range is given by chemotype where available; Tr—trace (<level of quantitation).

Compound	Chemotypes		Rage of Average Concentrations Reported per Chemotype (mg/g Dry Weight)	Reference
Agrospirol	I	I:	Tr–0.50	[45]
Alloaromandrene	I, II, III	I:	0.004–0.08	[53,60]
		II:	0.08–0.10	
		III:	0.05–0.10	
Aromadendrene	I	I:	0.02–0.13	[61]
α-Bisabolol	I, II, III	I:	Tr–1.10	[34,45,46,53,60,62,63,64]
		II:	0.57–1.22	
		III:	0.07–2.31	
α-Bisabolene	I, II, III	I:	0.13–0.50	[53,61]
		II:	0.11–0.29	
		III:	0.03–0.50	
β-Bisabolene	I, II, III	I:	0.05–0.17	[53]
		II:	0.18–0.51	
		III:	0.12–0.71	
Borneol	I, II, III	I:	0.01–0.03	[34,61,63,64]
		II:	0.05	
		III:	0.009–0.02	
α-bergamotene	I, II, III	I:	0.024–1.18	[34,53]
		II:	0.45–0.81	
		III:	0.018–0.68	
*Cis*-bergamotene	I, III	I:	0.07–0.11	[61]
		III:	0.21	
*Trans*-bergamotene	I, III	I:	0.12–0.28	[61]
		III:	0.04	
Bulnesol	I, II, III	I:	0.10–0.50	[34,45,53]
		II:	0.090–0.19	
		III:	0.070–0.49	
γ-cadinene	I, III	I:	0.41–0.60	[61]
		III:	0.02	
Camphene	I, III	I:	0.002–0.09	[34,60,63,64]
		III:	0.001–0.48	
Camphor	I	I:	0.001–0.01	[61,64]
P-Cimene	I, III	I:	0.016	[64]
		III:	0.01	
β-Caryophyllene	I, II, III	I:	0.24–8.20	[34,45,46,60,61,62,63,64,65]
		II:	0.86–3.90	
		III:	0.16–3.17	
β-Caryophyllene oxide	I, II, III	I:	0.005–0.06	[60,61,63]
		II:	0.02	
		III:	0.09	
*Trans*-β-caryophyllene	I, III	I:	0.02–0.06	[53,61]
		III:	0.06	
δ-3-carene	I, II, III	I:	Tr–0.60	[45,46,61,64,65]
		II:	Tr	
		III:	0.065–0.070	
α-Cedrene	I, III	I:	0.038	[64]
		III:	0.023	
β-Citronellol	I, III	I:	0.002	[60,64]
		III:	0.001–0.003	
α-curcumene	I, III	I:	0.008	[60]
		III:	0.017	
β -Curcumene	I, II, III	I:	0.014–0.61	[53,60]
		II:	0.061–0.16	
		III:	0.016–0.09	
Cyclounatriene	I, III	I:	0.02–0.13	[34]
		III:	0.086	
Elemene	I, II	I:	Tr–2.70	[45,65]
		II:	Tr	
γ-elemene	I, III	I:	0.104–1.89	[34,53,61]
		III:	0.04–0.068	
δ-elemene	I, III	I:	Tr–0.392	[34]
		III:	0.005	
Eucalyptol	II, III	II:	0.010–0.07	[53,60,63]
		III:	0.052–0.14	
Eudesma-3,7(11)-diene	I, III	I:	Tr–0.80	[34,61,65]
		III:	0.05	
Eudesmane	I, III	I:	0.33–0.55	[34]
		III:	0.04	
A-eudesmol	I, II	I:	0.02	[63]
		II:	0.26	
β-Eudesmol	I, II, III	I:	Tr–0.92	[45,53,61,63,64]
		II:	0.23–0.65	
		III:	0.085–1.01	
γ-Eudesmol	I, III	I:	Tr–0.80	[34,45,53,61]
		II:	0.30–0.78	
		III:	0.010–1.03	
α-farnesene	I, II, III	I:	0.02–0.06	[34,63]
		II:	0.24	
		III:	0.002	
β-farnesene	I, II, III	I:	0.019–1.96	[34,53,65]
		II:	0.73–1.6	
		III:	0.008–1.4	
*Trans*-β-farnesene	I, III	I:	0.31–1.06	[61,63]
		II:	0.35	
		III:	0.05	
Fenchone	I, II, III	I:	0.005–0.03	[60,63,64]
		II:	0.02	
		III:	0.007–0.008	
Fenchol	I, II, III	I:	0.047–1.09	[34,46,60,61,62,63,64]
		II:	0.09–0.31	
		III:	0.028–0.138	
Germacrene B	I, III	I:	0.25–1.27	[34]
		III:	0.34	
Geraniol	I, III	I:	0.01	[63,64]
		III:	0.004	
Geranyl Acetate	I	I:	Tr–0.70	[46]
Guaiol	I, II, III	I:	Tr–1.09	[34,45,53,61,63,65]
		II:	0.27–0.87	
		III:	0.010–1.21	
α-guaiene	I, III	I:	Tr–0.50	[45,65]
		II:	Tr	
		III:	Tr	
δ-guaiene	I, II	I:	Tr–0.80	[45,61,65]
		II:	0.8	
α-gurjunene	I	I:	0.1–0.46	[53]
Humulene	I, II, III	I:	Tr–4.00	[45,46,53,64]
		II:	0.64–1.11	
		III:	0.26–0.93	
α-Humulene	I, II, III	I:	0.09–1.93	[34,60,62,63,65]
		II:	0.32–0.36	
		III:	0.14–0.27	
Isopulegol	I, II	I:	0.02–0.04	[63]
		II:	0.02	
Ledene	I, II	I:	0.11–0.13	[63]
		II:	0.05	
Limonene	I, II, III	I:	Tr–9.1	[34,45,46,53,60,61,62,63,64]
		II:	0.079–1.14	
		III:	0.022–1.44	
Linalool	I, II, III	I:	Tr–3.10	[34,45,46,53,60,61,62,63,64]
		II:	0.27–0.35	
		III:	Tr–0.36	
*Cis*-linalool oxide	I, III	I:	0.002	[60]
		III:	0.005	
*Trans*-linalool oxide	I, III	I:	0.002	[60]
		III:	0.002	
Menthol	I, III	I:	0.001	[60]
		III:	0.001	
β-Myrcene	I, II, III	I:	0.12–14.8	[34,45,46,53,60,61,62,63,64,65]
		II:	0.20–3.02	
		III:	0.18–7.60	
Nerolidol	I, II, III	I:	0.02	[61]
		III:	0.01	
Trans-nerolidol	I, III	I:	0.019–1.66	[60,63,64]
		II:	0.09	
		III:	0.005–0.07	
β-Ocimene	I, III	I:	0.21–1.38	[34,53,63]
		II:	0.02	
		III:	0.19	
*Cis*-Ocimene	I, II, III	I:	0.006–3.9	[45,60,61,64,65]
		II:	1	
		III:	1	
*Trans*-Ocimene	I, III	I:	Tr–3.8	[46,60,64]
		III:	0.007–0.01	
α-phellandrene	I, II, III	I:	Tr–0.60	[65]
		II:	Tr	
		III:	Tr	
β-phellandrene	I, III	I:	Tr–2.1	[34,65]
		II:	0.7	
		III:	0.097–0.50	
α-pinene	I, II, III	I:	Tr–6.70	[34,45,46,53,60,61,62,63,64,65]
		II:	0.068–4.63	
		III:	0.004–1.40	
β-pinene	I, II, III	I:	Tr–2.00	[34,45,46,53,60,61,62,63,64,65]
		II:	0.054–0.80	
		III:	0.001–0.50	
α-phellandrene	I, II, III	I:	0.003–0.7	[46,60,61]
		II:	Tr	
		III:	0.001	
2-pinanol	I, III	I:	0.036–0.16	[34]
		III:	0.047	
Sabinene	I, III	I:	0.005	[60]
		III:	0.001	
*Cis*-sabinene hydrate	I, II	I:	0.015–0.08	[60,61,63]
		II:	0.003–0.03	
α-selinene	I, II, III	I:	0.04–1.36	[34,53,63]
		II:	0.26–0.65	
		III:	0.094–0.79	
β-selinene	I, II, III	I:	0.093–0.61	[53,63]
		II:	0.09–0.34	
		III:	0.10–0.22	
γ-selinene	I, II, III	I:	0.09–0.63	[53,61,65]
		II:	0.06–0.09	
		III:	0.03–0.14	
δ-selinene	I, III	I:	0.10–0.36	[34]
		III:	0.09	
Selina-3.7 (11) diene	I, II, III	I:	0.03–1.89	[53]
		II:	0.05–0.07	
		III:	0.06–0.092	
β-Sesquiphellanderene	I, II, III	I:	0.09–0.48	[53]
		II:	0.14–0.23	
		III:	0.074–0.19	
α-Terpinene	I, II, III	I:	Tr–0.10	[45,60,64]
		II:	Tr	
		III:	Tr–0.068	
γ-Terpinene	I, III	I:	0.02–0.06	[46,60,61,64]
		III:	0.01–0.06	
Terpineol	I, II, III	I:	Tr–0.70	[45]
		II:	0.6	
		III:	Tr	
Terpinen-4-ol	I, III	I:	0.02	[60]
		III:	0.01	
α-Terpineol	I, III	I:	0.04–0.9	[34,46,60,62,64,65]
		II:	0.29	
		III:	0.11–0.22	
Terpinolene	I, II, III	I:	Tr–13.9	[34,45,46,53,60,63,64,65]
		II:	0.010–3.70	
		III:	0.019–2.90	
Valencene	I, II	I:	0.001–0.06	[34,60,63]
		II:	0.01	
		III:	0.16	

## 4. Potential Roles of Secondary Terpenes

**Figure 2:**
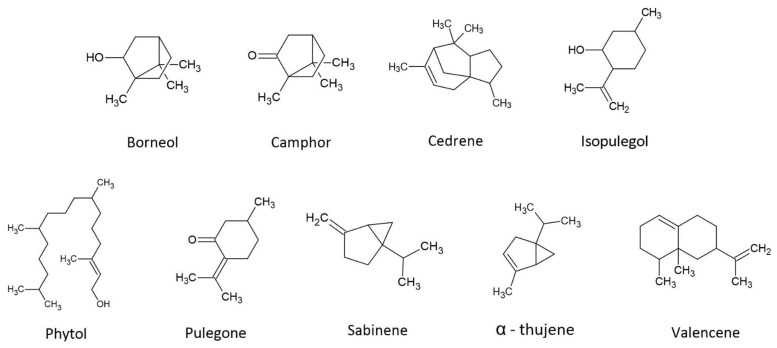
Structures of secondary terpenes present in *Cannabis sativa* L.

The biological activity of cannabis terpenes is a growing topic that been extensively covered in multiple reviews [13,23,66,67,68,69,70,71]. These reviews on the therapeutic properties of cannabis terpenes primarily cover the commonly encountered mono- and sesquiterpenes (e.g., β-caryophyllene, β-myrcene, α- and β-pinene, α-humulene, limonene, terpenoline, and linalool). For this reason, this review aims to further investigate nine secondary terpenes of cannabis, based on their abundance in the plant and their therapeutic potential (Figure 2). Regardless of their minor presence, the significant therapeutic value could point towards stronger or novel synergistic effects. The following is a summary of the more uncommon but notable secondary terpenes/terpenoids in cannabis and their potential therapeutic value; it is impossible to describe all the pharmacological effects of terpenes/terpenoids in this paper, but we shall give some examples of how these compounds possess multi-functional bioactivity. It is worth noting that the compounds often have multiple potential activities, and there is overlap of activities between terpene compounds.

### 4.1. Borneol

Identified as a monoterpene, borneol is a terpene derivative that can be found in several plant species, including *Cannabis sativa* L. [34,61,64]. The scent of this aromatic compound has been equated to a woody balsam aroma. Traditional Chinese medicine has employed the therapeutic properties of borneol for thousands of years as a resuscitation drug due to its active orifice-opening effects [72,73]. These effects are hypothesized to enhance blood–brain barrier (BBB) permeability [72,73,74], allowing for improved drug delivery to the central nervous system [73]. On top of its enhancement of BBB permeability, borneol also possesses anti-microbial, anti-inflammatory, anti-nociceptive, antithrombotic, neuroprotective, and genoprotective effects [75,76,77,78,79,80,81].

### 4.2. Camphor

This cyclic monoterpene ketone has been described as producing a strong mothball-like scent [82]. Like borneol, camphor has a long history of being used for its repellent and biological effects [83,84]. The medicinal properties of camphor oil include antibacterial, antiviral, antitussive, antimutagenic, anti-cancer, anti-inflammatory, antioxidant, and antidiabetic activity [85,86,87,88,89,90,91]. Studies evaluating camphor’s biological effects typically involve wood extracts of *Cinnamomum camphora*, the camphor laurel tree that primarily consists of high levels of camphor and its derivatives [92].

### 4.3. Cedrene

Cedrene is a sesquiterpenoid that is classified as a secondary terpene of cannabis as only small amounts of the terpenoid have been identified in select cultivars. This sesquiterpenoid is commonly found in cedar and juniper trees [93,94]. The aroma produced by cedrene has been described as a woody, crisp scent. Like many aromatic compounds, the biological activities of cedrene have been explored primarily through extracts of cedarwood oil, which has been characterized as having copious amounts of the sesquiterpenoid [95]. Although many of the studies report the effects of full cedarwood oil, cedrene is a primary constituent of this oil and thus leads to speculation that the biological activity of cedarwood oil is due to cedrene, with activities including antifungal, anti-microbial, and anti-cancer [96,97,98]. A few studies have been performed on isolated α-cedrene, suggesting potential anti-obesity properties [99,100].

### 4.4. Isopulegol

Identified as a monoterpene alcohol, isopulegol can be found at different concentrations in a variety of plants, including lemongrass, mint, eucalyptus, and several others [101,102,103]. The scent of isopulegol has been described as a minty fragrance [104]. Because of its presence in a diversity of plants, researchers have described its potential bioactivity, including antidepressant [105], antianxiety [105], anticonvulsant [106], gastroprotective [107], and anti-inflammatory activity [108]. Though isopulegol is said to contain several diverse bioactive properties, more research is required to characterize the mechanism in play.

### 4.5. Phytol

Phytol is a diterpenoid that has been described as having a grassy-fresh aroma [104]. Phytol is a common terpenoid of highly aromatic plants such as green tea, mint, tarragon, basil, and cannabis cultivars [109,110]. This terpenoid has been speculated to hold antioxidant [111,112], anti-inflammatory [113], analgesic [112], anti-cancer [111,114] anti-anxiety [115], anti-convulsant [116], and sedative [117] properties. Phytol and its derivatives have also been explored for toxicity in immune-compromised mice, suggesting non-toxic effects [118,119,120].

### 4.6. Pulegone

As a monoterpene, pulegone can be found in various aromatic herbs, but is commonly associated with the mint family, such as catnip and peppermint [121,122]. Known for its minty fragrance, pulegone has been identified at low concentrations in cannabis [22,123]. Researchers have suggested that pulegone contains anti-microbial [124], anti-anxiety [125], antipyretic [126], sedative [126,127], and anti-inflammatory [128] properties.

### 4.7. Sabinene

This bicyclic monoterpene can be found in a variety of different plant species and is often associated with a spicy flavor and aroma. Cannabis cultivars typically contain small concentrations of sabinene; however, some cultivars have been characterized as having more sabinene than others, such as Super Silver Haze and Arjan’s Ultra Haze [104]. The medical benefits of this monoterpene suggest anti-inflammatory [129], antioxidant [130], and anti-microbial [131] properties. The known benefits of this terpene are limited, requiring more research and exploration of the effects of sabinene in cannabis.

### 4.8. Thujene

Like many monoterpenes, thujene can be found in a variety of plant-derived essential oils such as eucalyptus [132], frankincense [133], and dill [134]. Similar to humulene, α-thujene produces a woody, spicy aroma [135]. Researchers have tested the bioactivity of essential oils that consist of high levels of thujene, such as the essential oil of *Boswellia serrata*, which has been reported to consist of 61.36% α-thujene [136]. Investigations exploring the bioactivity of essential oils containing α-thujene suggest antioxidant [137], anti-inflammatory [138], anti-microbial [139], and analgesic [140] properties. Although these studies provide insight into the bioactivity of essential oils containing this compound, more research is required to delineate the therapeutic properties of isolated α-thujene.

### 4.9. Valencene

The sesquiterpene valencene produces an aroma that is often associated with citrus fruits such as Valencia oranges [141]. In cannabis, valencene has been reported in several different cultivars, but only at low concentrations [70]. Aside from its appealing scent, valencene’s bioactivity has been explored through various essential oil profiles, speculating anti-inflammatory [142], neuroprotective [143], anti-allergic [144], and anti-microbial [145] properties.

**Figure 3:**
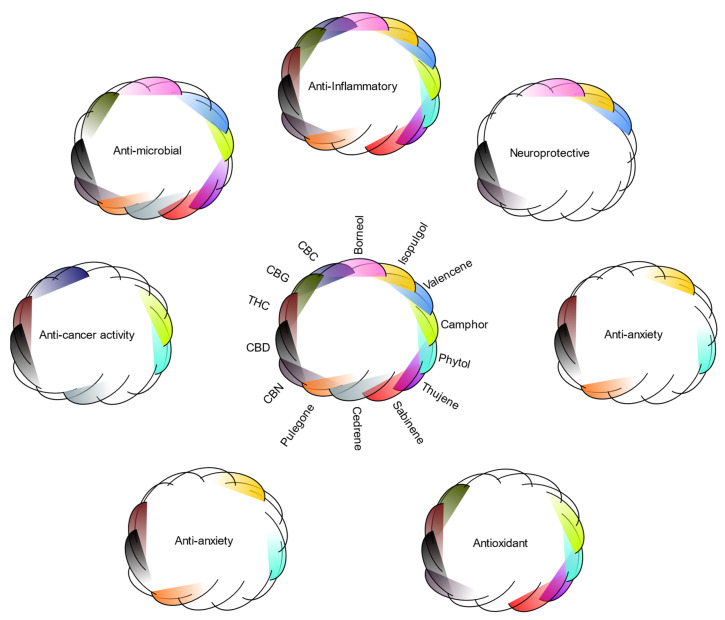
Bioactivities of the nine secondary terpenes covered in this review, along with cannabis’s principal cannabinoids (CBD, cannabidiol; THC, tetrahydrocannabinol; CBG, cannabigerol; CBN, cannabinol; CBC, cannabichromene). Bioactivity circles are color-coded to match the legend in the middle; the presence of a particular shading in the circle is indicative that the terpene or cannabinoid has been reported to possess that bioactivity. The figure demonstrates that not only do terpenes have multiple potential bioactivities, but different compounds possess overlapping activities, suggesting their potential to exert combination effects.

While minor terpenes may not be the most abundant in cannabis, they have the potential to aid in the biological activities of cannabis. These terpenes demonstrate overlapping activity with each other, often targeting the same biological function (even if mechanism of action remains unknown) (Figure 3). Likewise, an overlap of therapeutic benefit between cannabinoids and these secondary terpenes may be inferred based on current cannabinoid research [60], suggesting a potential to increase the efficacy of these cannabinoids in an additive or synergistic manner.

## 5. Mechanism of Action for Terpenes—Pharmacologic Receptor Targets (TRPs)

Several studies have investigated the pharmacodynamics of the receptors for the major terpenoids found in cannabis (e.g., β-caryophyllene, β-myrcene, β-pinene, α-humulene, linalool). For instance, β-caryophyllene has been found to be an agonist at the cannabinoid receptor 2 (CB2), peroxisome proliferator-activated receptor gamma (PPARγ), and the toll-like receptor 4 (TLR4)/CD14/MD2 complex, while β-myrcene is an agonist at α2-adrenergic receptors and transient receptor potential cation channel subfamily V member 1 (TRPV1) [13,146,147,148,149]. The information on the receptors modulated by the minor terpenes found in cannabis is much more variable and will be the focus of the discussion below.

Borneol is an agonist of TRPM8. This activation of TRPM8 by borneol has been found to be temperature sensitive and dose-dependent across a range of concentrations, from 10 µM to 2 mM; however, no EC_50_ was reported because the study failed to reach a maximal response [150,151]. The activation of TRPM8 receptors by borneol has been found to activate glutamatergic and GABAergic transmission in the spinal cord, leading to anti-nociceptive activity [152,153]. The activation of TRPM8 by borneol has also been shown to enhance the chemosensitivity of lung cancer cell lines to doxycycline [151]. Borneol is also an agonist of TRPV3 (EC_50_ = 3.45 mM) channels [154]. Furthermore, borneol is an antagonist of the TRPA1 channel, with an IC_50_ of 0.2–0.3 mM in cell-based assays [155,156]. The activation of TRPV3 and inhibition of TRPA1 also likely contribute to the antinociceptive properties of borneol. Of note, the antagonist/agonist profile of borneol at these receptors matches that of several cannabinoids, including CBD, CBG, and THC; however, these effects occur at relatively high levels.

Camphor is a major terpenoid constituent of cannabis but is best known as an isolate from the camphor laurel (*Cinnamomum camphora*). This compound is FDA-approved as an additive to soothing creams and ointments and as a component of over-the-counter respiratory treatments. Camphor has been found to act as an agonist at TRPM8 and TRPV3 and an antagonist at TRPA1, which is perhaps not surprising considering the structural similarity of camphor to borneol. Despite this structural similarity, camphor is less potent at both TRPV3 (EC_50_ = 6.03 mM) and TRPA1 (IC_50_ = 1.26 mM) compared to borneol [154,156]. At TRPM8, camphor has an EC_50_ of approximately 4.5 mM [157]. Additionally, camphor has been shown to be a partial agonist at TRPV1, with similar potency as at TRPV3 and TRPA1 (EC_50_ > 3 mM) [158,159]. The action of camphor at these receptors likely accounts for its analgesic activities. In addition, activation of the TRPV family of receptors has been linked to the ability of camphor to relax the trachea in rats, which may help explain its anti-congestive activities [160].

Cedrene has been identified as a potent agonist of the olfactory receptor 10J5 (OR10J5), a GPCR that is also found in liver and muscle tissue [161]. In human hepatocytes, cedrane has been shown to lower lipid levels through OR10J5. Furthermore, cedrane has been shown to reduce muscle atrophy induced by a high fat diet in mice, this action is mediated through the mouse ortholog of OR10J5, MOR23 [162]. This study also found that cedrane increased muscle mass and strength, possibly through increasing expression of IGF1.

Isopulegol has been identified as an agonist of the most abundant GABA_A_R in the brain, α1β2γ2, with an EC_50_ of approximately 3.25 µM. Activation of the GABAR produces sedative effects, and these receptors are targets for both analgesics and anticonvulsant medications [163]. Isopulegol is also an agonist at TRPM8 and may also antagonize the TRPV1 receptor [164,165]. Either of these actions may account for the anti-nociceptive properties of isopulegol that have been described in mice [165].

Phytol and its metabolites can act as natural ligands for a variety of transcription factor receptors. This list includes the peroxisome proliferator-activated receptor (PPAR) α and γ; however, an EC_50_ was not reported because the assay did not reach a plateau at 100 µM, the highest concentration tested [166,167]. Additionally, phytol has been shown to be an agonist of retinoid X receptors (RXR), with EC_50_ estimates ranging from 41.9 to 67.2 µM, depending upon the isotype [168]. Through activation of these receptors, phytol has been shown to reduce cancer cell viability in a number of cancer cell lines. Indeed, it has been found to have a lower IC_50_ in the lung adenocarcinoma cell line, A549, than the chemotherapeutic agent methotrexate [169]. Phytol induced apoptosis in this system through the activation of the TNF receptor, TRAIL, and FAS. Additionally, the authors used molecular docking to suggest that phytol may bind to glucose-6-phosphate dehydrogenase to inhibit tumor progression. In vitro, phytol has also been shown to increase the release of CA^2+^ reserves via activation of GPR40, a G-protein-coupled receptor that normally binds to free fatty acids, with an EC_50_ of 34.5 µM [170]. The activation of PPARs, RXRs, and GPR40 by phytol may also be of potential therapeutic benefit for the treatment of diabetes, and because of the ability to activate RXR receptors, phytol is also being pursued by the cosmeceutical industry as an anti-aging treatment in lieu of retinol (which is not well tolerated by all individuals due to its activation of TRPV1) [171,172].

Using a recently developed in vitro receptor binding assay, pulegone was shown to be the component in *Ziziphora clinopodioides* that binds and potentially activates β_1_-adrenoceptors [173]. Pulegone has been found to be an agonist of avian TRPM8 at low concentrations; however, it antagonizes this receptor at higher concentrations [174]. This study also found that pulegone is an antagonist of TRPA1 at both low and high concentrations. Taken together, these data suggest that pulegone may have anti-nociceptive and analgesic utility.

Computer-based molecular docking research predicted that sabinene may be a potent interactor with L-asparginase from the bacterial pathogen, *Salmonella typhimurium* [175]. This study found that sabinene had a higher docking score than the antibiotic ciprofloxacin, suggesting that sabinene may have antibacterial properties and may be a good candidate for antibiotic development. Additionally, sabinene has been found to reduce levels of the inflammatory marker nitric oxide in cells exposed to lipopolysaccharide [129]. Another molecular docking study suggested that sabinene may interact with the spike protein on the SARS-CoV2 (COVID-19) virus along with three cell membrane proteins (transmembrane serine protease 2, cathepsin B, and cathepsin L) that play a role in mediating viral entry into cells [176]. Additional studies will be needed to determine if any of these interactions occur in vivo, as well as which receptors might mediate the decrease in nitric oxide production caused by sabinene. An in silico study suggested that thujene may have a modest binding affinity for the SARS-CoV2 main protease and papain-like protease, but further work will be needed to confirm these findings [177]. Additionally, no studies could be located that identified potential human receptors for this terpene.

Valencene has been reported to be cardioprotective following myocardial infarction in rats, and this protection is mediated through the inhibition of the NF-κB pathway, oxidative stress, and cardiac hypertrophy; however, the receptors that mediate this inhibition were not examined [178]. Valencene has been found to be an antagonist of the calcium ion channel TRPV1 and the slow release calcium release-activated calcium channel protein 1 (ORAI1), which inhibited the melanin content in UVB exposed melanoma cells, and may therefore be useful for treating photo-aging of the skin [179]. This inhibition, may also mediate the ability of valencene to potentially treat atopic dermatitis [180].

## 6. Synergy and the Entourage Effect: Beyond Cannabinoids

Natural product discovery efforts are traditionally reductionist in nature, devoted to condensing a complex botanical extract down to a single bioactive agent for drug development purposes. This is true for cannabis research and development, where the single molecule approach remains the dominant approach [181]. However, botanical medicines, including cannabis, are in fact complex diverse concoctions of phytochemicals that have the potential of exerting differing and potentially complementary biological effects. Indeed, it is often observed that these mixtures work in concert to achieve a specific physiological effect [182]. Compounds can work in a synergistic manner, in which each active compound potentiates the other to achieve a greater than expected benefit when combined (i.e., 1 + 1 > 2). If one compound, having no activity of its own, impacts the efficacy of an active molecule to increase activity (i.e., 1 + 0 > 1), this is known as an entourage effect [26]. As cannabis research has evolved, there has been a growing body of evidence that cannabinoids beyond THC demonstrate efficacy in humans [183,184] and that synergy/entourage could potentially play a large role in the bioactivity of cannabis extracts and products [22,185].

Botanical synergy and entourage have been demonstrated in cannabis, first in the combination of THC with other, “minor”, cannabinoids. Johnson et al. (2010) tested a cannabis-based extract for patients with intractable pain and found that, while the THC dominant extract did not improve patient outcome versus the placebo (the mean pain Numerical Rating Scale (NRS) was a nonsignificant change of −1.01 vs. −0.69), a whole plant extract (the only difference being the presence of CBD) demonstrated a significant improvement in pain outcome (mean NRS of −1.37 vs. −0.69) compared to the placebo [186]. Animal studies focusing on analgesia also evidenced greater response from a full-spectrum cannabis extract as compared to pure CBD dosing [187]. Recently, experiments with a seizure mouse model looked at the effects of different strains of cannabis that all contained an equivalent CBD concentration. While all were effective, there were noticeable differences between the strains, and profiling 94 phytocannabinoids across 36 of the most commonly used *Cannabis* plants prescribed to patients in Israel led to the conclusion that these other cannabinoids have an impact on the overall efficacy of cannabis plant extracts [188]. In one in vitro study, one study of breast cancer cell lines revealed that the extract of the whole cannabis was more effective than a preparation featuring THC by itself; the boost in activity was attributed to the presence of “minor” cannabinoids cannabigerol (CBG) and tetrahydrocannabinolic acid (THCA) [189]. Complex fractions from cannabis extracts demonstrated synergistic interactions on colorectal cancer cell lines [190].

Cannabinoids have been widely studied for the treatment of epilepsy [191,192]; complex extracts containing multiple cannabinoids were found to treat severe epilepsy, such as Dravet and Lennox-Gastaut syndromes, at lower doses than trials using purer preparations (e.g., Epidiolex, which contains 97% CBD) [66,193]. A 2018 meta-analysis by Pamplona et al. of 11 studies demonstrated that the response rate at 50% improvement of seizure frequency was similar between the two groups, but the average daily doses were significantly different: 27.1 mg/kg/d for purified CBD as opposed to 6.1 mg/kg/d. for cannabis extracts [194]. Moreover, the incidence of adverse events was discernably higher in the CBD versus complex extract treatments (*p* < 0.0001), a result that the authors attributed to the lower dose utilized, which was achieved in their opinion by the synergistic contributions of other entourage compounds.

Most synergy studies have focused primarily on the interactions between cannabinoid structures, despite the fact that the original definition of the entourage effect arose from the interaction of 2-acyl-glycerol esters with cannabinoids [26]. As terpenes are a large and diverse family of phytochemicals found in cannabis, they have the potential to serve as potentiating agents working in concert with cannabinoids. Terpenes, broadly speaking, have been found to be broadly synergistic, helping modulate the activity of a number of other botanicals, pharmaceuticals, and compounds. The terpenes highlighted in this review also have a strong history of synergistic activity with other compounds. Borneol was shown to synergize with curcumin to induce apoptosis in human melanoma cells [195], potentiate the activity of berberine and baicalein in inhibiting in vitro and in vivo fungal growth [196], and function as a potentiating agent to sensitize cancer cells to doxorubicin treatment [151]. Sabinene [197] and pulegone [198] each indicated the potential for synergistic interactions with prescription antibiotics in treating bacterial infections, and β-caryophyllene and phytol demonstrated combination effects inducing apoptosis in skin epidermoid cancer cells [199]. Terpenes have also demonstrated efficacy in the treatment of mood and anxiety disorders, suggesting the possibility of combination effects with cannabinoids for more effective treatments [200]. Thus, while the potential of cannabis terpenes to possess additive or synergistic properties was originally posited as hypothetical based upon similar bioactivities [23], more recent studies have explored this possibility in earnest.

A 2021 study by LaVigne et al. found that α-humulene, geraniol, linalool, and β-pinene were cannabimimetic at the CB_1_ receptor and produced cannabinoid-like behaviors in a mouse model. Furthermore, the terpenes potentiated the effects of a cannabinoid agonist, suggesting synergistic activity [201]. Di Giacomo et al. treated triple negative breast cancer cells (MDA-MB-468) with hemp inflorescences and pure compounds of CBD, caryophyllene and cannabichromene. The presence of these other compounds induced the potentiating effects of CBD, likely mediated through CB2 activation [202]. However, separate studies observed that none of the terpenes α-pinene, β-pinene, β-caryophyllene, linalool, limonene, and β-myrcene were found to alter potassium channel signaling in AtT20 cells expressing CB_1_ and CB_2_ receptors, and did not interact with THC at the receptor [203], nor did they affect changes in intracellular calcium at the human transient receptor potential ankyrin 1 (hTRPA1) or human transient receptor potential vanilloid 1 (hTRPV1) channels [204]. Using a radioligand ([^3^H]-CP55,940) to measure binding at the CB1 and CB2 receptors, none of the tested terpenes (myrcene, α-pinene, β-pinene, β-caryophyllene, and limonene) had interactions with receptors, nor did they modulate the binding of THC or CBD [205]. Similarly, no synergy was detected between myrcene and CBD in modulating inflammation and analgesic properties in a rat adjuvant monoarthritis model [206]. Research on colorectal cancer cells did not detect any enhancement of activity when terpenes were included as part of a complex CBD oil compared to the effect of CBD alone [207].

The divergence of results involving potential synergy or entourage effects has led to doubt surrounding the entourage effect in cannabis and whether it really holds pharmaceutical potential. Cogan (2020) references several studies where individual cannabinoids did not improve the clinical performance of THC or CBD [208]. However, the intellectual leap to label cannabis potential synergistic interactions as “questionable” is perhaps premature. Botanicals or combinations exhibiting synergy or entourage does not necessitate that the effects take place at the same target to elicit a heightened response; compounds can exhibit “pharmacodynamic synergism” by acting at multiple cellular targets (seen in both antibiotic and cancer synergistic therapies) [209,210] and “pharmacokinetic synergism” by increasing the solubility or disposition (absorption, distribution, metabolism) of active constituents [211,212], and can limit side effects of the active constituent [213,214] or disrupt resistance mechanisms [215,216]. Indeed, the study Santiago et al. that purported the “absence of entourage” nevertheless suggested that synergy could still be taking place at a different molecular target than the CB receptors [203]. Therefore, as further studies are developed, it would be prudent to also employ phenotypic assays that encompass more than a single receptor/enzyme/target and can better deduce the combination effects at complementary sites and pathways to deliver heightened results. Thus, the heightened skepticism or dismissal of synergism in cannabis is perhaps unwarranted at this time, as there exists a growing body of evidence suggestive that not only do multiple cannabinoids work in concert to produce heightened effects (or potentially lower deleterious side effects), as seen in the prescription drugs that utilize multi-component cannabis extracts (Sativex^®^ and Epidolex^®^) [186], but that terpenes/terpenoids can also potentially function as synergists with cannabinoids to deliver amplified results. Furthermore, studies from our own group, using an animal model of chemotherapeutic induced peripheral neuropathy, demonstrated an enhanced effect at reducing mechanical hypersensitivity by an extract containing equal parts CBG and CBD, in addition to other cannabinoids and terpenes, compared to pure CBG [217]. Additionally, in the same animal model we found that pure CBD was without an effect; however, when animals were treated with a complex hemp extract at the same CBD concentration, a reduction in mechanical sensitivity was observed [218]. These studies suggest that there is a potential interaction between cannabinoids and terpenes that can enhance the effect of pure cannabinoids alone. Moreover, the lack of synergy in vitro may not hold true for results in intact organisms, and the entourage effect does not have to be present or absent in all systems or biomedical indications. The search for synergy and entourage effects within the diverse phytochemical landscape of cannabis remains in its infancy; to better understand these combination effects, further research on the potential combination effects of cannabis’s polypharmacy is essential to establish mechanisms of interaction, cellular targets of interest, and adverse events.

## 7. Conclusions and Future Directions

While commonly thought of as a psychoactive plant producing one of the most famous mind-altering chemicals discovered by humans, cannabis is a biosynthetic engine, producing hundreds of diverse phytochemicals that have the potential to impact a wide variety of human health conditions. In particular, cannabis produces 200 terpene structures that are of interest, both as independently bioactive molecules as well as by modulating or potentiating the effects of cannabinoids or other phytochemicals from cannabis. Terpenes are already widely implemented in traditional medicines and pharmaceuticals, as well as in industrial processes, perfumery, cosmetics, and food additives. They demonstrate generally low toxic profiles and high bioavailability and are highly selective to TRP channels, among other targets. There are known cannabimimetic activities of some terpenes, and they already have shown synergy amongst each other in other in vitro and in vivo studies. Thus, there is a firm foundation for cannabis synergy and the involvement of terpenes in the flavor, aroma, and bioactivity of cannabis. Investigations into potential combination effects in cannabis is a growing field, one which requires rigorous experimental design and execution but has the possibility to evolve our understanding of cannabis’s diverse pharmaceutical effects.
